# Armed conflict, child marriage, and maternal healthcare utilization: Evidence from 82 surveys in 49 low-and lower-middle-income countries

**DOI:** 10.1017/S1463423626100899

**Published:** 2026-02-27

**Authors:** Risha Singh, Srinivas Goli, Shubhra Kriti, Anu Rammohan

**Affiliations:** 1 College of Public Health, University of Nebraska Medical Center, Omaha, USA; 2 Fertility and Social Demography, IIPS: International Institute for Population Sciences, Mumbai, India; 3 Population Studies, IIPS: International Institute for Population Sciences, Mumbai, India; 4 Department of Economics, The University of Western Australia, Perth, Australia

**Keywords:** armed conflicts, child marriage, low and low-middle income countries, maternal health care

## Abstract

**Aim:**

To examine whether the association between child marriage and maternal healthcare utilization differs between conflict and non-conflict settings, and whether armed conflict amplifies the negative effects of child marriage on maternal healthcare utilization.

**Background:**

Armed conflicts hinder progress in reproductive and maternal health, particularly in low- and lower-middle-income countries, by weakening health systems, disrupting access to care, and increasing gender-based vulnerabilities. Child marriage, which is common in such contexts, may further limit women’s ability to seek adequate maternal healthcare. While both conflict exposure and child marriage are known to adversely affect maternal health outcomes, evidence on their intersection remains limited. Understanding their combined influence is essential for designing effective primary healthcare and humanitarian interventions.

**Methods:**

We used data from 82 Demographic and Health Surveys (1994–2020) across 49 countries, linked spatially and temporally with armed conflict information from the Uppsala Conflict Data Program. The sample included 452,192 women aged 15–49. Maternal healthcare utilization was measured using continuum-of-care indicators: at least one antenatal care (ANC) visit, four or more ANC visits, four or more ANC visits with institutional delivery, and four or more ANC visits with institutional delivery and postnatal care (PNC). Associations were estimated using binomial logistic regression models, with robustness checks including interaction effects, macro-level analyses, and mediation analyses.

**Findings:**

Women married before age 18 had significantly lower odds of utilizing maternal healthcare compared to those married at 18 or older. These disparities were strongest in conflict-affected areas, where child brides consistently showed the lowest utilization of ANC, institutional delivery, and PNC. Maternal education, household wealth, urban residence, and media exposure partially mitigated these associations. Additional analyses confirmed the robustness of findings across alternative model specifications, conflict measures, and subgroups.

## Introduction

Improving reproductive and maternal health (RMH) care outcomes has been a longstanding global health priority and one of the Sustainable Development Goals (SDGs). However, despite significant improvements in the reduction of maternal mortality rates, it continues to remain a major concern, especially in developing countries. According to the World Health Organization’s data on maternal mortality, nearly 800 women have lost their lives from preventable pregnancy and childbirth-related causes every single day in 2020. Of these, 95% of the maternal deaths took place in low and lower middle-income nations. Moreover, 86% of the global maternal deaths were concentrated in Sub-Saharan Africa (70%) and South Asia (16%) (WHO *et al*., 2023). Among the various factors that affect maternal health outcomes, armed conflicts in low and middle-income countries is one that poses a great challenge to achieve the United Nations SDGs, related to gender equality (Goal 3) and violence against children (Goal 16), by 2030. The impacts of armed conflicts on public health (Østby *et al*., 2018; Carpiniello, 2023; Le and Nguyen, 2023; Arage *et al*., 2024; Badanta *et al*., 2024; David and Eriksson, 2025; Nikiema and Kafando, 2025; Salem *et al*., 2025), not only through direct deaths and injuries but also through indirect pathways, are well established (Li and Wen, 2005; Grimard and Laszlo, 2014; Keasley *et al*., 2017; Salazar *et al*., 2018; Wagner *et al*., 2019; Garry and Checchi, 2020). Conflicts often lead to the destruction of healthcare systems and infrastructure, and thereby, the discontinuation of various healthcare services. Maternal and reproductive healthcare services are among the most affected components of basic healthcare services during armed conflict situations (Bosmans *et al*., 2008; Chi *et al*., 2015; Chukwuma and Ekhator-Mobayode, 2019).

Maternal health outcomes, however, are not only linked to availability but are also invariably associated with the accessibility or utilization of various healthcare services. According to Ghobarah *et al*. (2003:191), ‘health conditions are shaped by the interplay of exposure to conditions that create varying risks of death and disease for different groups in society and the ability of groups in society to gain access to health care and receive the full range of benefits produced by the health-care system’. Further, research has shown that women’s maternal health care utilization can be severely affected during armed conflicts (Namasivayam *et al*., 2017; Chukwuma and Ekhator-Mobayode, 2019). Furthermore, there are individual-level factors alongside other socio-economic factors that determine the utilization of maternal healthcare. Child marriage is among these crucial factors that adversely influence maternal healthcare services, inclusive of lower utilization of antenatal care visits (ANC), institutional delivery, and skilled birth attendance during childbirth (Kamal and Ulas, 2022).

Child marriage is defined by UNICEF as ‘any formal marriage or informal union between a child under the age of 18 years and an adult or another child’. It may be regarded as a violation of children’s human rights and is also considered a form of gender-based violence. Research on child marriages in conflict situations shows that the practice has been used as a protective measure to ensure the safety and security of women during a crisis. However, child marriage is considered as a harmful practice as it is detrimental to women’s health from multiple aspects. Previous research has also shown the negative impacts of gender-based violence on the uptake of various health-care services, including RMH care (Sekine and Carter, 2019; Dadras *et al*., 2022).

Existing literature predominantly examines armed conflict’s impact on maternal health or child marriage’s effect on maternal healthcare in isolation, with limited research investigating their synergistic effects. A critical gap remains in understanding how conflict zones exacerbate maternal healthcare barriers for child brides – through disrupted health systems, displacement, or heightened gender inequalities. This study tests the hypothesis that conflict-related increases in child marriage rates indirectly impair maternal health outcomes by restricting healthcare access. Addressing this intersection could inform targeted humanitarian interventions for vulnerable adolescent mothers in crisis settings. Our study attempts to bridge this research gap by analysing the association between child marriages and maternal healthcare utilization in conflict versus non-conflict situations. Maternal healthcare utilization of women in our study has been assessed using outcome indicators such as antenatal care visits, institutional delivery, and post-natal care. This study demonstrates that child brides experience poorer maternal healthcare utilization outcomes than women married at 18+ years, particularly in conflict zones. The mediation analyses suggest that although a major part of poor maternal health outcomes is driven by conflict, a portion of it is also channelled through child marriage. Our findings contribute to the literature by examining how armed conflict exacerbates the negative effects of child marriage on maternal healthcare access. The analysis reveals that child marriage’s detrimental impact on healthcare utilization is most pronounced in conflict-affected areas where early marriage is more prevalent.

## Background

Previous literature on armed conflicts and health care has extensively documented the exposure to armed conflicts and its adverse implications on the health and well-being of an individual throughout their life-course (Iqbal, 2006; Williams *et al*., 2012; Urdal and Che, 2013; Kesternich *et al*., 2014; Lee, 2014; Lindeboom and van Ewijk, 2015; Keasley *et al*., 2017; Kadir *et al*., 2019; Garry and Checchi, 2020; Arage *et al*., 2024; Badanta *et al*., 2024; Nikiema and Kafando, 2025). The ramifications, however, are not gender neutral across the population, as previous research has shown that women are more prone to conflict-related adversities compared to men (Ghobarah *et al*., 2003; Plümper and Neumayer, 2006). Not only are they likely to suffer from long, drawn-out indirect impacts of conflicts, owing to their biological differences, but also due to their different social and gender roles (Moser and Clark, 2001; Petchesky, 2008; Buvinic *et al*., 2013; Meagher *et al*., 2021).

For instance, one of the case studies conducted among Syrian refugees to understand child marriage in the context of armed conflict showed how conflict and displacement can significantly increase rates of child marriage and simultaneously limit access to maternal healthcare (Alking, 2019).

I spent with my mother and little sister many nights scared; we were alone since my father died during the shelling of our city, and many teenagers in the camp harassed us. Marriage brokers in the camp offered my mother to marry her minor daughter to a man in his thirties for a thousand dollars, which my mother strongly rejected, but as harassment increased, my mother agreed (Alking, 2019: 42).

The participants also highlighted a lack of autonomy in terms of health care-related decisions as well as experiencing intimate partner violence, leading to serious health issues.

My son got sick once, and I could not call my husband. We waited until the next day for my husband to return home. I feared that my husband would be angry if he went to the hospital without his okay (Alking, 2019: 43).

I was in pain, I went to the hospital to find out the cause of that pain, and I knew it was infections, the doctor recommended us not to have sex for a while, but my husband did not abide and forced me to have sex causing hysterectomy due to infections (Alking, 2019: 45).

Importantly, RMH concerns for women in armed conflicts are a major concern, as women are primarily responsible for reproduction and child care activities (Inter-agency Working Group on Reproductive Health in Crises, 2010). The concerns are exacerbated with increased insecurity when women migrate or are forcibly displaced, lose their spouse or family members; lose their jobs, income, and assets; or face morbidity or injury as a result of armed conflicts. Further, armed conflicts also destroy or alter the capacities of individuals or women to cope with the adversities, as armed conflicts lead to the destruction of familial, social, economic and legal structures, including health infrastructures and welfare provisions (Kottegoda *et al*., 2008). The effect of armed conflict on health, especially for women, is evident from previous research. For instance, Bendavid *et al*. (2021) in their study found that women of child-bearing age living within 50 kilometres of an armed conflict have three times higher mortality risk relative to women in peaceful settings. Additionally, Jawad *et al*. (2021) estimated that there were 0.3 million excess maternal deaths between 2000 and 2019 that might be attributable to conflicts. Similarly, using a dataset on a sample of 35 African countries, Wagner *et al*. (2019) showed that between 1990 and 2016, mortality among women increased by 112 deaths per hundred thousand-person years within 50 km of their residence due to conflicts.

High maternal mortality in armed conflicts, as mentioned by Urdal and Che (2013), is possibly channelized through three mechanisms: (1) increased unskilled abortions and pregnancies due to dysfunctional health systems and lack of skilled medical personnel and emergency obstetric care, (2) heightened risk of morbidity and mortality because of forced migrations and shortage of medical care, and (3) a greater risk of malnutrition during or post pregnancy because of low access to nutrition as an outcome of the loss of jobs, wages and assets. So, inarguably, one of the important factors implicated in negative maternal and reproductive health is access to various healthcare facilities and services. It can also be corroborated from the findings of Bashour *et al*. (2021) based on a qualitative study of women requiring childbirth and delivery care in conflict-affected Syria. They highlight that accessing a hospital to receive maternal healthcare was one of the major concerns as described by Syrian women, along with a lack of availability of specialized medical personnel and facilities, due to the conflicts. Similarly, in a conflict-stricken Northern Mali, women faced considerable difficulties in accessing much-needed medical care, even in cases of emergency, owing to the looting and devastation of health facilities by the warring groups (Degni *et al*., 2015). In Nigeria, Chukwuma and Ekhator-Mobayode (2019) showed that access to antenatal, institutional and skilled delivery, and maternal health care was lower because of the Boko Haram Insurgency. Further, mobility restrictions, curfews, and closures reduced the accessibility of RMH services among Palestinian women because of the longstanding Palestine’s humanitarian crisis (Giacaman *et al*., 2007; Bosmans *et al*., 2008).

**Figure 1.**
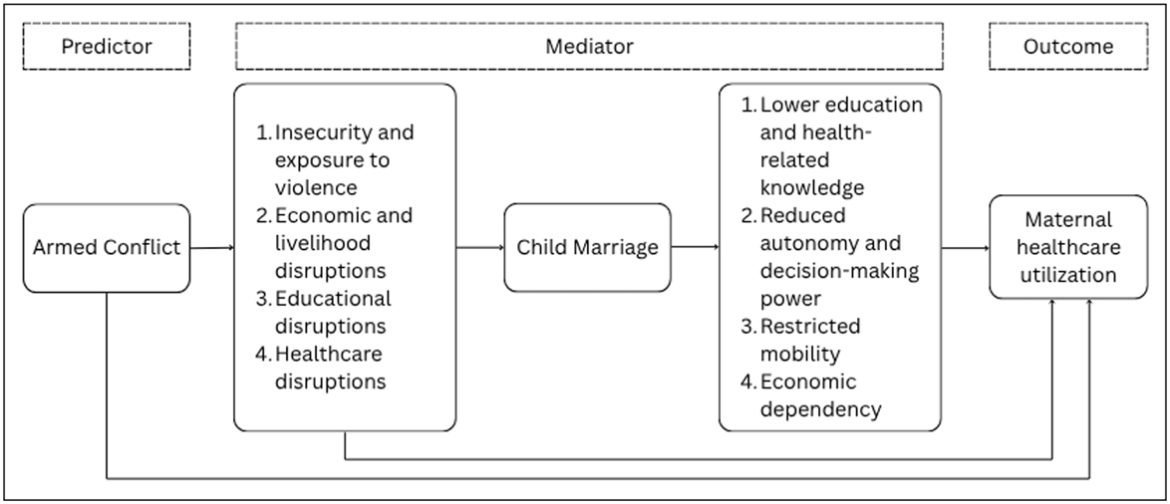
Conceptual framework. *Source*: Author’s conceptualization based on synthesis of prior studies.

Importantly, access to the maternal and reproductive health services is effectively moderated by women’s education, knowledge, and wealth (Mayhew *et al*., 2008; Siziya *et al*., 2009; Karkee *et al*., 2013), distance to the health facility (Dhakal *et al*., 2011; Choulagai *et al*., 2013), utilization of at least one antenatal care (Sharma *et al*., 2014), especially, autonomy over health-related decisions (Kempe *et al*., 2013) and other socio-demographic factors (Dhaher *et al*., 2008; Shrestha *et al*., 2012; Adam *et al*., 2014; Gopalan *et al*., 2017). Given the multiple and interrelated mechanisms through which armed conflict can shape women’s RMH outcomes, it is important to clearly articulate the pathways examined in this study. Drawing on prior literature and the theoretical rationale discussed above, we developed a conceptual framework illustrating both the direct effects of conflict exposure on maternal healthcare utilization and the indirect pathway operating through child marriage, as shown in Figure 1.

## Methodology

### Data

Our analysis is primarily based on data from three major sources: (a) Uppsala Conflict Data Program (UCDP), (b) Demographic and Health Survey (DHS) Datasets, and (c) World Development Indicators (WDI).

The UCDP is one of the prominent data providers on organized violence or armed conflicts and civil war, comprising data for four decades, which includes information on battle-related deaths along with their location.

The DHS includes comparable individual-level data on marriage, reproductive, maternal health, and other socio-economic background indicators for women, along with information on their location at the provincial level across the countries used for the study.

The WDI compiles data mainly on development indicators from internationally recognized statistics and national official sources to provide the most recent and reliable estimates at the regional, national, and international levels.

For the analysis, we combined datasets from UCDP and DHS. Information from DHS on women residing in a province was spatially matched with conflict data of the same province from UCDP. Considering that the DHS does not provide GPS coordinates for all countries included in this study, we relied on provincial-level identification for both conflict events and DHS households. We harmonized official province names across countries and survey years to ensure that each respondent’s province was accurately matched with the corresponding UCDP conflict events occurring in the same administrative unit. Temporally, we utilized the information on the conception year (calculated using their child’s date of birth information), of the last-born child in 5 years prior to the DHS survey, and the DHS interview year; and the start and end years of conflicts from the UCDP data. Upon matching the cases temporally and spatially, we assigned every woman the information on total battle-related deaths occurring in any conflict event during their child’s conception year till the year of DHS survey interview, along with other socio-economic information.

**Figure 2.**
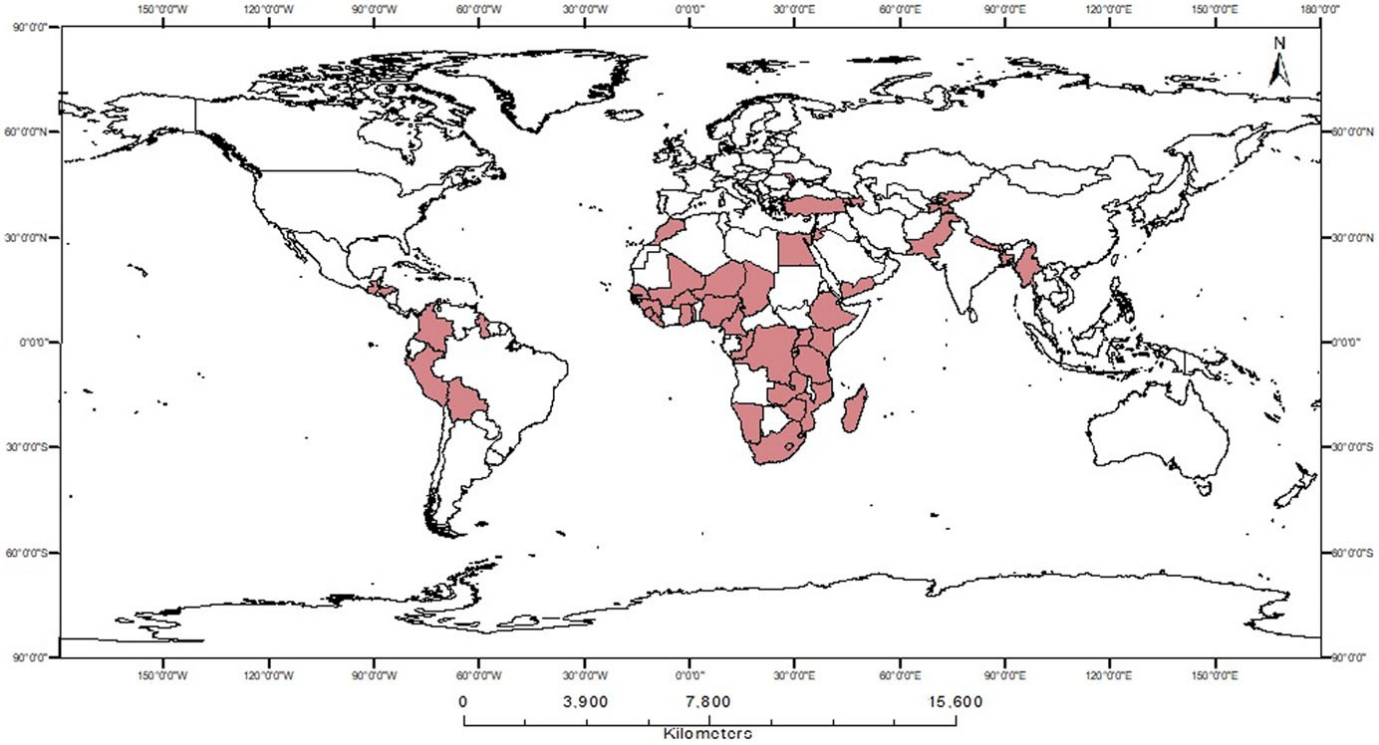
Selected countries in the sample. *Source*: Mapped by authors based on information Uppsala Conflict Data Program.

After merging the data from various sources, the cases with missing data for multiple study variables were dropped. However, given the large sample size and the relatively small proportion of missing data on primary variables, the overall impact on representativeness is likely to be minimal. Our final sample includes 452,192 women aged 15 to 49 years from 82 DHS surveys spread over a total of 523 provinces in 49 countries from 1994 to 2020. Figure 2 presents the countries selected in our sample. For further details on the number of cases in individual countries across different regions, refer to Appendix Table A1.

### Variables

The study variables were categorized as outcome, predictor, and control variables. For the analysis, the outcome measure, i.e., maternal health access, was assessed through four variables that capture maternal healthcare access or utilization in a continuum (Rammohan *et al*., 2021). It includes (a) at least one ANC, (b) Four or more ANC, (c) Four or more ANC and institutional delivery, and (d) Four or more ANC and institutional delivery as well as post-natal care (PNC).

The two main predictor variables for our study are child marriage and armed conflict. Child marriage has been captured using the variable, age at marriage, assigned with a value of 1 if a woman reported being married before 18 years of age, and 0 otherwise.

We measured armed conflict using two specifications: (a) Conflict exposure, a binary indicator (1 = Exposed to at least one conflict, 0 = Otherwise), (b) Conflict intensity, a continuous variable, calculated as a total number of battle-related deaths that occurred between the earliest and latest conflict event in the women’s province of residence during their child’s conception year until the interview year divided by total number of years in that period. The indicator was further segmented into terciles (Intensity_1, Intensity_2, and Intensity_3); and 0 (Intensity_0) otherwise.

We controlled for the individual and household’s socio-economic and demographic characteristics using mother’s age, age at first birth, education, media exposure; child’s sex, birth order, multiple birth, household size, household head’s age and sex, residence (rural/urban), wealth index, percentage of households in poorest quintile at a provincial level, country and year dummies.

Table 1 shows the summary statistics of the study variables used for the analysis. Approximately 68.1% of our sample was exposed to at least 1 conflict while 32% were never exposed to any conflict. Nearly half (46.2%) of women in our sample were married below 18 years of age. With regards to maternal healthcare utilization, nearly three-fourths of the total women in our sample had at least one ante-natal care visit, while slightly just above one-fourth (26.5%) of them had received full care. 51% of the sampled women had a male child, while 49% of them had a female child. Median age at first birth for the women of our sample was 19 years. Education-wise, 34.4% of women in our sample had no education, 27% had primary education, 30.4% of women had completed secondary education, and just 8.1% of women had completed higher secondary education or above. A majority of women in our sample (75.2%) were found to have media exposure. The study sample is predominantly rural, with 68.9% contribution to the total sample.

**Table 1.** Descriptive statistics of the study variables

Factor type	Variables	Average/ proportion (SE)
Armed conflict	Conflict exposure	
	Exposed to at least 1 conflict	68.09 (0.001)
	Never exposed to conflict	31.91 (0.001)
	Conflict intensity (Mean)	89.83 (0.007)
Child marriage	Child marriage status	
	Married below 18 years	46.21 (0.002)
	Married at 18 years or above	53.79 (0.002)
Maternal health	Maternal healthcare utilization	
	At least 1 ANC	76.34 (0.001)
	4 or more ANC	48.54 (0.001)
	4 or more ANC and institutional delivery	31.75 (0.001)
	4 or more ANC and institutional delivery and PNC	26.52 (0.001)
Child characteristics	Child’s sex	
	Male	51.22 (0.002)
	Female	48.78 (0.002)
	Multiple birth	
	Yes	1.44 (0.000)
	No	98.56 (0.000)
	Birth order	
	First	23.09 (0.001)
	Second	22.03 (0.001)
	Third	16.58 (0.001)
	Fourth and above	38.30 (0.002)
Mother characteristics	Mother’s age	
	Below 25	30.33 (0.001)
	25–35	47.46 (0.002)
	35 and above	22.21 (0.001)
	Age at first birth (median)	19 (0.001)
	Education	
	Illiterate	34.45 (0.001)
	Primary	26.99 (0.001)
	Secondary	30.43 (0.001)
	Higher secondary & above	8.14 (0.000)
	Any media exposure	
	Yes	75.21 (0.001)
	No	24.79 (0.001)
Household characteristics	Household head’s sex	
	Male	88.25 (0.001)
	Female	11.75 (0.001)
	Household size	
	1–3	11.70 (0.001)
	4–6	46.46 (0.001)
	7 and above	41.84 (0.002)
	Residence	
	Rural	68.86 (0.001)
	Urban	31.14 (0.001)
	Wealth quintile	
	Poorest	23.19 (0.001)
	Poorer	21.64 (0.001)
	Middle	20.21 (0.001)
	Richer	18.82 (0.001)
	Richest	16.15 (0.001)
	Percentage of households in poorest quintile at the provincial level	
	0–9%	22.38 (0.001)
	10–19%	30.79 (0.001)
	20–29%	25.89 (0.001)
	30% and above	20.95 (0.001)
Total observations	*N*	452,192

### Econometric strategy

Firstly, using the two variables: child marriage and conflict exposure, the sample was divided into four distinct groups: (a) Women who were married below 18 years and were exposed to conflict (child marriage = 1 & conflict exposure = 1), (b) Women who were married at 18 years and above and were exposed to conflict (child marriage = 0 & conflict exposure = 1), (c) Women who were married below 18 years and not exposed to conflict (child marriage = 1 & conflict exposure = 0), and (d) Women who were married at 18 years and above and not exposed to conflict (child marriage = 0 & conflict exposure = 0).

Our main analysis compares maternal health outcomes for these four groups of women. The comparison assessed the role of child marriage on maternal health indicators in conflict versus non-conflict zones (the distinction between conflict and non-conflict zones was made based on exposure to armed conflict), using the variable Conflict Exposure. Women with a value 1 for the variable ‘Conflict Exposure’ were considered to be in a conflict zone, 0 if they resided in a non-conflict zone.

Bivariate tables and graphs are used to show basic relationships between our variables of interest. Further, we used a Binomial Logistic regression model with odds ratios (OR) to estimate the net association between child marriage and maternal health indicators in conflict versus non-conflict zones, while controlling for confounding factors. We used two generalized Binomial Logistic equations given as (Retherford and Choe, 2011):
(1)

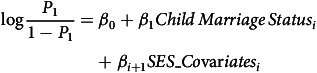



(2)

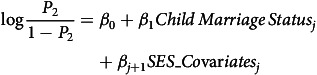




In Equations 1 and 2, 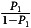 and 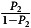 represent the odds for our maternal healthcare utilization variables for women in conflict and non-conflict zones, respectively, with 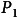 and 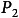 being the probability for a specific outcome in the respective zones. 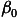 is the intercept. 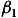 is the coefficient for the predictor variables 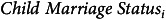 representing the child marriages in conflict zones in Equation 4.1, and 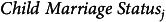 representing the child marriages in non-conflict zones in Equation 4.2. 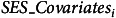 and 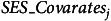 are the socio-economic control variables in the respective equations. We have compared 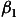 from Equations 1 and 2 to access the association between child marriages and maternal health utilization in conflict versus non-conflict zones.

Survey sampling weights provided by DHS were de-normalized to adjust for differential population sizes across the countries and applied to all descriptive and regression analyses to ensure nationally representative estimates. Standard errors were estimated using cluster-robust variance estimators at the province level, which is the unit at which conflict exposure is measured. Clustering at this level accounts for shared unobserved characteristics and spatial correlation among respondents exposed to the same conflict environment. As an additional robustness check, we also estimated models with country-level clustering, which yielded similar results.

## Results

### Descriptive statistics

**Figure 3.**
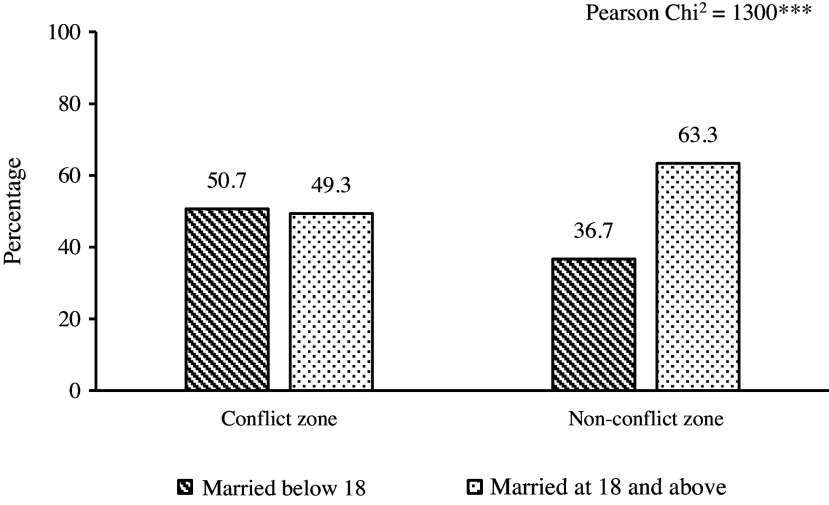
Prevalence of Child Marriage by the Conflict Status (*N* = 452,192). *Source*: Author’s calculation using UCDP and DHS datasets.

Figure 3 shows the distribution of women in our sample by the status of their child marriage (married below age of 18 years or at 18 years and above) in conflict and non-conflict zones, respectively. It shows that more than half of the women who lived in areas of armed conflicts were married before 18 years of age (50.7%), which was slightly higher than women living in non-conflict areas (49.32%). Contrastingly, child brides (36.7%) are nearly half of the women married at 18 years or above in non-armed conflict areas (63.3%). Further, the chi-squared test of independence also shows a statistically significant relationship between the variables.

**Figure 4.**
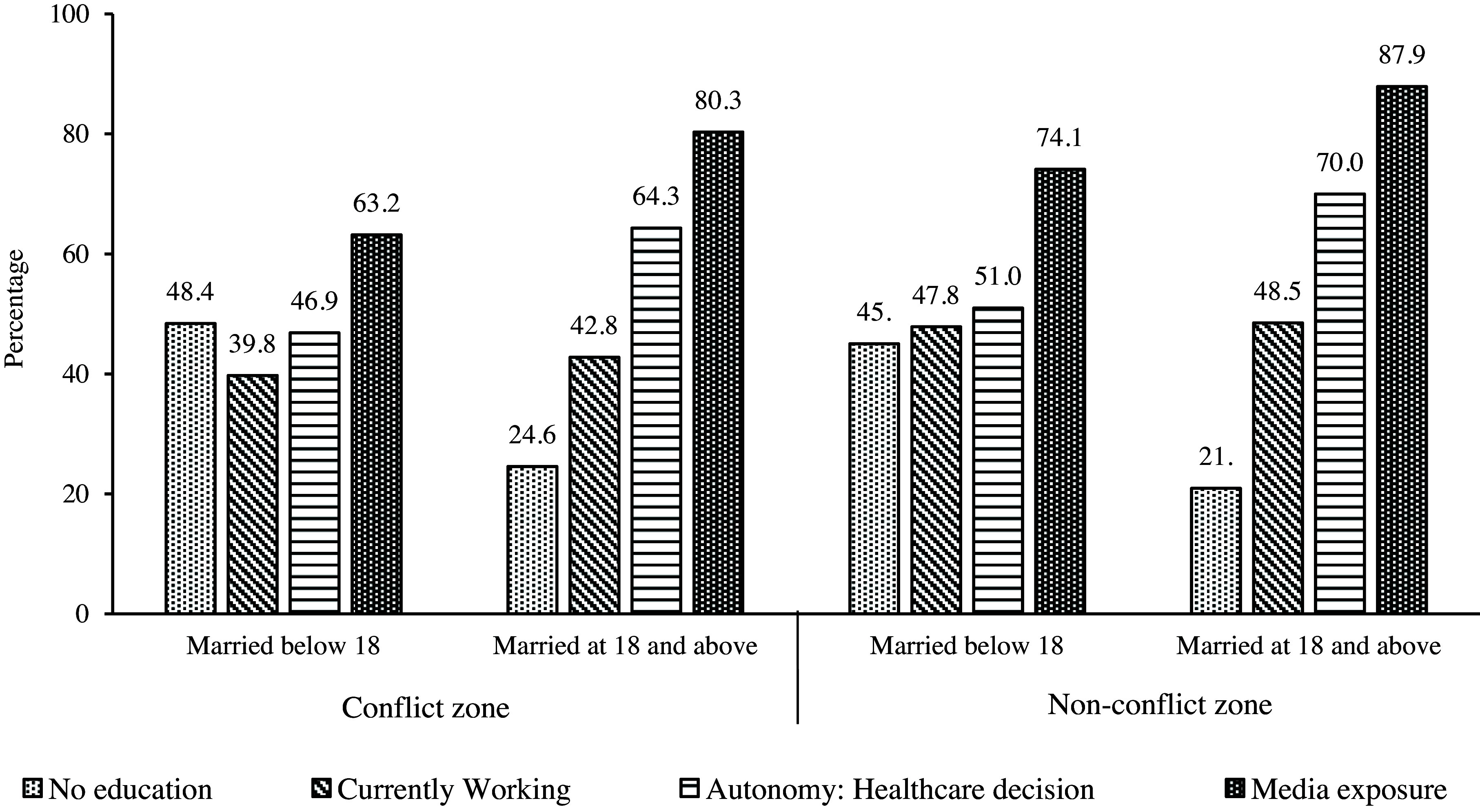
Socio-economic status of the sampled women by their child marriage status in conflict versus non-conflict zones (*N* = 452,192). *Source*: Author’s calculation using UCDP and DHS datasets.

Not only the prevalence of child marriage varied, but their background characteristics are also different for women residing in conflict and non-conflict zones. As shown in Figure 4, women married below 18 years in conflict areas had the highest share of no education and the lowest share of currently working women compared to others. Unsurprisingly, these women also have the least exposure to mass media and the lowest autonomy in healthcare decisions.

**Figure 5.**
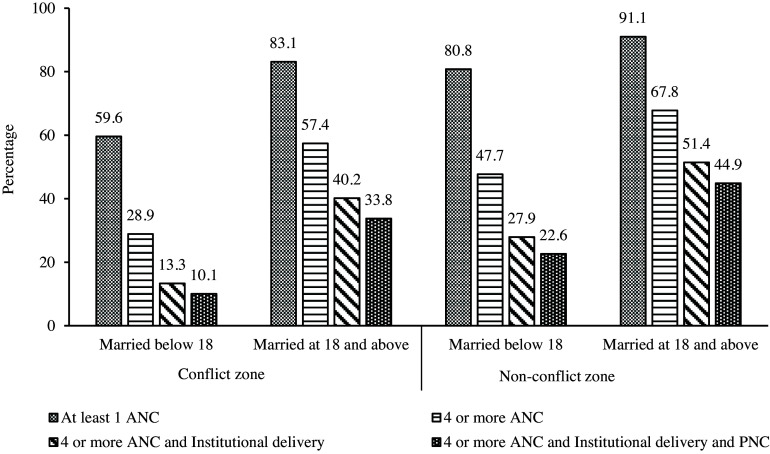
Reproductive and maternal health indicators for women by their child marriage status in conflict versus non-conflict zones (*N* = 452,192). *Source*: Author’s calculation using UCDP and DHS datasets.

Figure 5 shows that overall, child brides in both the zones had lower health care utilization compared to those married above 18 years of age. Importantly, child brides from conflict zones had the lowest health care utilization while women from non-conflict zones married at 18 and above had the highest healthcare utilization, across all indicators. Specifically, full maternal healthcare utilization was less than half and one-fourths among the child brides in conflict zone (10.1%) compared to child brides (22.6%) and women married at 18 and above (44.88%) in non-conflict zones respectively.

### Binomial logistic regression results

Table 2 presents the estimates of the Binomial logistic regression model showing the association between child marriage and RMH indicators based on conflict exposure. The results show that, after controlling for socio-economic, individual and demographic characteristics, child brides have lower odds of receiving the RMH continuum-of-care compared to women married at age 18 years or above. Additionally, women married below 18 years of age from conflict zones have even lower odds for all the four indicators of RMH care (OR: 0.85, 0.89, 0.89, and 0.88 respectively, *p* < 0.01) compared to those from non-conflict zones (OR: 0.85, 0.97, 0.94, and 0.96 respectively, *p* < 0.01). Further, the odds of receiving maternal health care were higher (OR = 1.05 for conflict zones and OR = 1.03 for non-conflict zones, *p* < 0.01) if the child was male (compared to female), or if multiple births (compared to a single birth).

**Table 2.** Logistic regression estimates: odds ratios showing effects of child marriage on reproductive and maternal health indicators in conflict versus non-conflict zones (*N* = 452,192)

	Conflict zone					Non-conflict zone			
	At least 1 ANC	4 or more ANCs	4 or more ANCs & institutional delivery	4 or more ANCs & institutional delivery & PNCs		At least 1 ANCs	4 or more ANCs	4 or more ANCs & institutional delivery	4 or more ANCs & institutional delivery & PNCs
Child marriage	0.849*** (0.016)	0.888*** (0.014)	0.894*** (0.016)	0.879*** (0.017)		0.847*** (0.016)	0.971** (0.012)	0.942*** (0.012)	0.964*** (0.013)
Child is male	1.025* (0.013)	1.016 (0.012)	1.043*** (0.014)	1.048*** (0.015)		1.011 (0.014)	1.007 (0.009)	1.028*** (0.010)	1.031*** (0.010)
Multiple births	1.444*** (0.082)	1.436*** (0.069)	1.913*** (0.102)	1.911*** (0.107)		1.537*** (0.087)	1.329*** (0.046)	1.758*** (0.064)	1.711*** (0.063)
Child’s birth order	0.903*** (0.009)	0.879*** (0.007)	0.799*** (0.008)	0.814*** (0.009)		0.858*** (0.008)	0.841*** (0.006)	0.768*** (0.006)	0.799*** (0.006)
Mother’s age	1.003 (0.002)	1.014*** (0.001)	1.021*** (0.002)	1.023*** (0.002)		0.999 (0.002)	1.016*** (0.001)	1.021*** (0.001)	1.019*** (0.001)
Mother’s age at 1st birth	1.009*** (0.003)	1.007*** (0.002)	1.017*** (0.003)	1.013*** (0.003)		1.007** (0.003)	1.004** (0.002)	1.010*** (0.002)	1.015*** (0.002)
Mother completed primary education	2.196*** (0.053)	1.781*** (0.037)	1.893*** (0.047)	1.856*** (0.052)		2.167*** (0.051)	1.400*** (0.020)	1.408*** (0.022)	1.363*** (0.022)
Mother completed secondary education	3.619*** (0.107)	2.514*** (0.060)	2.807*** (0.073)	2.691*** (0.074)		3.611*** (0.113)	1.959*** (0.036)	2.175*** (0.041)	1.991*** (0.038)
Mother completed higher secondary education	8.334*** (0.569)	4.817*** (0.179)	6.540*** (0.240)	5.627*** (0.210)		7.153*** (0.702)	3.311*** (0.127)	3.566*** (0.124)	2.883*** (0.089)
Household head’s age	1.001* (0.001)	1.003*** (0.001)	1.004*** (0.001)	1.004*** (0.001)		0.999** (0.001)	1.000 (0.000)	1.001* (0.000)	1.001** (0.000)
Household head is male	0.960 (0.024)	0.969 (0.019)	0.938*** (0.020)	0.931*** (0.021)		0.969 (0.024)	0.978* (0.013)	0.966** (0.014)	0.961*** (0.014)
Household size	0.991*** (0.003)	0.976*** (0.002)	0.973*** (0.003)	0.974*** (0.003)		1.004* (0.002)	0.995*** (0.001)	0.993*** (0.002)	0.993*** (0.002)
Rural	0.710*** (0.025)	0.774*** (0.018)	0.704*** (0.018)	0.739*** (0.019)		0.724*** (0.022)	0.901*** (0.015)	0.677*** (0.013)	0.741*** (0.014)
Poorer	1.265*** (0.031)	1.201*** (0.027)	1.312*** (0.033)	1.400*** (0.040)		1.305*** (0.028)	1.254*** (0.019)	1.473*** (0.025)	1.456*** (0.025)
Middle	1.779*** (0.051)	1.579*** (0.038)	1.771*** (0.049)	1.945*** (0.060)		1.552*** (0.040)	1.484*** (0.025)	1.859*** (0.036)	1.846*** (0.036)
Richer	2.219*** (0.073)	2.028*** (0.056)	2.529*** (0.076)	2.766*** (0.091)		1.972*** (0.066)	1.817*** (0.035)	2.444*** (0.053)	2.420*** (0.052)
Richest	3.481*** (0.155)	3.128*** (0.108)	4.277*** (0.155)	4.660*** (0.182)		3.821*** (0.192)	2.643*** (0.065)	3.811*** (0.102)	3.666*** (0.098)
Media exposure	1.655*** (0.035)	1.501*** (0.028)	1.531*** (0.035)	1.614*** (0.043)		1.555*** (0.032)	1.250*** (0.019)	1.282*** (0.022)	1.294*** (0.023)
% of households in poorest quintile at provincial level	0.995*** (0.001)	0.995*** (0.001)	0.987*** (0.001)	0.990*** (0.001)		0.993*** (0.001)	0.997*** (0.001)	0.994*** (0.001)	0.995*** (0.001)
Country dummies	Yes	Yes	Yes	Yes		Yes	Yes	Yes	Yes
Year dummies	Yes	Yes	Yes	Yes		Yes	Yes	Yes	Yes
Observations (*n*)	179160	179160	178962	178255		273030	273030	273030	272557

*Note*: Standard errors in parentheses. *p*-values: * *p* < 0.1, ** *p* < 0.05, *** *p* < 0.01.

Overall, an increase in mother’s age (OR = 1.02 for conflict zones and OR = 1.01 for non-conflict zones, *p* < 0.01), age at first birth (OR = 1.01 for conflict zones and OR = 1.02 for non-conflict zones, *p* < 0.01) increased the odds of receiving antenatal, institutional delivery and postnatal care. Importantly, higher maternal education attainment significantly increased the likelihood of receiving full care (OR = 1.86 to 5.63 in conflict zones and OR = 1.02 to 2.88 in non-conflict zones, *p* < 0.01) compared to mothers with no education. Residence-wise, rural women were less likely to access full care (OR = 0.74 in both conflict zones and non-conflict zones, *p* < 0.01) relative to urban women. Also, higher media exposure (OR = 1.61 in conflict zones and OR = 1.29 in non-conflict zones, *p* < 0.01) and belonging to households from wealthier households (Poorer to richest: OR = 1.40 to 4.67 in conflict zones and OR = 1.46 to 3.67 in non-conflict zones, *p* < 0.01) is positively associated with better odds of receiving the maternal health care.

### Robustness checks

We test the robustness of our results for alternative specifications as described below:

**(a) Binomial Logistic Regression with Interaction Effects**

We re-estimate a binomial logistic regression model including an additional interaction term for conflict and child marriage, to check the association between armed conflict exposure and child marriage, controlling for conflict intensity, on maternal health utilization (At least one ANC, four or more ANC, four or more ANC and Institutional Delivery, and four or more ANC with Institutional Delivery and PNC). The equation used for analysis is given as (Retherford and Choe, 2011):
(3)

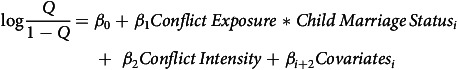




In Equation 3, 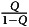 represent the odds for our maternal health utilization outcomes. 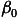 is the intercept. 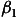 is the coefficient for the predictor variable, which is an interaction term of conflict exposure and child marriage status. 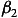 is the coefficient of 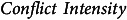 and 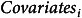 are the variables representing other socio-economic background factors included as controls.

Table 3 presents the odds ratios for the interaction effect of the armed conflict and child marriage on maternal healthcare utilization indicators. Firstly, the coefficient for the interaction term is statistically significant, indicating that the association between child marriages and maternal healthcare utilization depends on their exposure to armed conflict. It also illustrates that the size of the β coefficient for the interaction term decreased and was the smallest for full maternal healthcare (OR = 0.82, *p* < 0.01). An OR of 0.82 for institutional delivery among child brides in conflict-affected areas suggests an 18% reduction in odds, which is programmatically significant when applied across large populations. In low-resource settings, even modest percentage-point drops can translate into thousands of missed facility births or antenatal visits, increasing the risks of maternal morbidity and mortality. The coefficients for individual terms, child marriage, and conflict exposure, however, were not statistically significant. These results indicate that the likelihood of women married as children to access full care was significantly lower, especially when exposed to armed conflicts.

**Table 3.** Logistic regression estimates: odds ratios for interaction effects of conflict exposure and child marriage status on reproductive and maternal health indicators

	At least 1 ANC	4 or more ANCs	4 or more ANCs & institutional delivery	4 or more ANCs & institutional delivery & PNCs
No conflict exposure × child marriage (Ref.)				
Conflict exposure × child marriage	0.987 (0.022)	0.853*** (0.014)	0.869*** (0.016)	0.824*** (0.016)
Married at 18 and above (Ref.)				
Child marriage	0.856*** (0.015)	0.995 (0.011)	0.966*** (0.012)	0.994 (0.013)
No conflict exposure (Ref.)				
Conflict exposure	1.045 (0.061)	1.071 (0.044)	1.015 (0.052)	1.062 (0.053)
Conflict_intensity0 (ref.)				
Conflict_intensity1	0.771*** (0.043)	0.967 (0.042)	1.086 (0.057)	1.069 (0.055)
Conflict_intensity2	0.961 (0.055)	1.016 (0.043)	0.975 (0.051)	1.012 (0.051)
Conflict_intensity3	0.875** (0.054)	0.802*** (0.038)	0.733*** (0.042)	0.855*** (0.048)
Child is male	1.018* (0.009)	1.010 (0.007)	1.032*** (0.008)	1.037*** (0.008)
Child is multiple birth	1.484*** (0.059)	1.366*** (0.039)	1.809*** (0.055)	1.770*** (0.054)
Child’s birth order	0.883*** (0.006)	0.856*** (0.005)	0.781*** (0.005)	0.807*** (0.005)
Mother’s age	1.001 (0.001)	1.015*** (0.001)	1.020*** (0.001)	1.020*** (0.001)
Mother’s age at 1st birth	1.008*** (0.002)	1.005*** (0.001)	1.013*** (0.002)	1.015*** (0.002)
Mother completed primary education	2.191*** (0.039)	1.540*** (0.019)	1.562*** (0.022)	1.520*** (0.022)
Mother completed secondary education	3.619*** (0.080)	2.187*** (0.032)	2.397*** (0.037)	2.234*** (0.036)
Mother completed higher secondary education	8.137*** (0.457)	4.153*** (0.112)	5.099*** (0.134)	4.053*** (0.099)
Household head’s age	1.000 (0.000)	1.001** (0.000)	1.002*** (0.000)	1.002*** (0.000)
Household head is male	0.965** (0.017)	0.978* (0.011)	0.957*** (0.012)	0.954*** (0.012)
Household size	0.997* (0.002)	0.990*** (0.001)	0.988*** (0.002)	0.988*** (0.002)
Rural	0.708*** (0.017)	0.845*** (0.012)	0.684*** (0.011)	0.741*** (0.011)
Poorer	1.284*** (0.021)	1.228*** (0.015)	1.411*** (0.020)	1.422*** (0.021)
Middle	1.662*** (0.032)	1.506*** (0.021)	1.815*** (0.028)	1.848*** (0.031)
Richer	2.088*** (0.048)	1.884*** (0.030)	2.468*** (0.042)	2.518*** (0.045)
Richest	3.508*** (0.116)	2.827*** (0.058)	4.032*** (0.087)	4.048*** (0.090)
Media exposure	1.627*** (0.025)	1.365*** (0.016)	1.370*** (0.019)	1.389*** (0.020)
% of households in poorest quintile at provincial level	0.994*** (0.001)	0.996*** (0.000)	0.991*** (0.000)	0.993*** (0.000)
Country dummies	Yes	Yes	Yes	Yes
Year dummies	Yes	Yes	Yes	Yes
Observations	452192	452192	452192	452192

*Note*: Standard errors in parentheses. *p*-values: * *p* < 0.1, ** *p* < 0.05, *** *p* < 0.01.

Further, the odds of receiving full care were significantly lower for women facing the highest intensity of conflicts (OR = 0.86, *p* < 0.01) relative to women facing low intensity of conflicts (OR = 1.069). Further, the likelihood of accessing full care was 1.52 to 4.05 times higher for women with any education than for those with no education. Likewise, women from wealthier households (comparing across the five wealth quintiles from poorest to richest) have better odds (OR = 1.42 to 4.05, respectively, *p* < 0.01) of accessing full maternal healthcare compared to the women from the poorest wealth quintile. Further, better media exposure was significantly associated with higher odds (OR = 1.39, *p* < 0.01) of utilizing full care relative to those without any media exposure.

**(b) Macro-level Analysis**

We also assessed the robustness of our findings by examining the relationship between child marriage, conflict and maternal healthcare utilization at the macro-level, using ‘country’ as a unit of analysis. The dataset for macro-level analysis was compiled using datasets primarily from UCDP, DHS, WDI, and Multiple Indicator Cluster Surveys (MICS). Based on the availability of data, the analysis was conducted for the period 1994 to 2020. The entire time period was divided into six separate time periods: 1994–1998, 1999–2003, 2004–2008, 2009–2013, and 2014–2020, owing to the data availability of chosen indicators at a mean interval of 4 years rather than for every year. In case of multiple data points for a select period, the mean average value has been considered.

The outcome measure was Maternal mortality ratio (MMR), and the main predictor variable was an interaction term of armed conflict and child marriage. Armed conflict was captured in terms of its intensity, calculated as total battle-related deaths per 1000 population in a country in a given time period, while child marriage was captured using the child marriage rate, as defined by the United Nations.

The control variables include the log of Gross Domestic Product per capita (constant), Poverty Headcount Ratio, Female Secondary School Gross Enrolment Ratio and Rural Population (as a percent of the total population). We also included log of Neighbouring Conflict Intensity to control for the intensity of conflict in a neighbouring country. Cultural factors were further controlled using regional and time dummy variables to account for the potential unobserved social and cultural norms. Table A2 provides the detailed definitions of all the variables used for micro analysis, and Table A3 shows the descriptive statistics for these variables.

The fixed effects regression model has been used with the interaction effects to assess the association of the interaction term of armed conflict and child marriage with MMR.
(4)

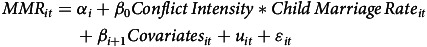




In Equation 4, 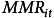 the outcome variable indicates the MMR for country 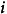 at time 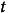 . 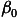 is the coefficient for the predictor variable, 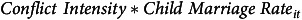 , an interaction term of armed conflict and child marriage. 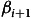 is the coefficient for 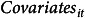 , which represents the socio-economic and other control variables, 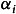 is the intercept, 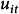 is within-entity error term and 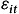 is overall error term.

Table 4 presents estimates for the interaction of armed conflicts and child marriage at the macro-level on MMR. Overall, the interaction between child marriages and armed conflicts is significantly associated with MMR. Model 1 shows the net effects without the inclusion of the interaction term, while Model 2 includes the interaction term. After the inclusion of the interaction terms, there is a statistically significant association between the interaction term and a single-unit increase in the child marriage rate and battle-related deaths per 1000 population, cumulatively attributing to 9.14 units (calculated as 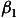 + 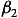 + 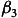 ) increase in MMR. Even after controlling for other background socio-economic indicators (as shown in Model 2 to Model 8), the interaction term continues to be statistically significant. For instance, in Model 8; comprising all the potential controls, a one-unit increase in the child marriage rate and conflict intensity is associated with a 0.22-unit increase in the MMR.

**Table 4.** Fixed-effects regression model estimates: association of interaction between armed conflicts and child marriages on reproductive and maternal health indicator (Maternal mortality ratio), 1994–2018

Factor type	Variables	Gross effects	Net effects						
		Model 1	Model 2	Model 3	Model 4	Model 5	Model 6	Model 7	Model 8
Interaction	Conflict intensity × child marriage	0.13*** (0.04)	0.12*** (0.04)	0.10*** (0.04)	0.09 (0.07)	0.28*** (0.05)	0.14*** (0.04)	0.19*** (0.06)	0.19*** (0.06)
Child marriage	Married below 18	11.19*** (0.89)	7.71*** (0.95)	7.27*** (0.96)	5.87*** (1.28)	7.94*** (1.07)	7.48*** (0.96)	7.50*** (1.06)	7.31*** (1.05)
Armed conflict	Conflict intensity	−2.18** (0.97)	−2.11** (0.93)	−1.49 (0.94)	−1.33 (3.32)	−9.95*** (2.20)	−2.41*** (0.92)	−6.46*** (2.38)	−7.28*** (2.36)
	Neighbouring conflict intensity	–	0.27*** (0.07)	–	–	–	–	–	0.34*** (0.08)
Economic	GDP per capita constant (log)	–	–	−2.56 (19.85)	–	–	–	−2.13 (21.59)	−5.57 (21.28)
	Poverty headcount Ratio	–	–	–	1.63*** (0.57)	–	–	–	–
Social	Female secondary GER	–	–	–	–	−1.40*** (0.40)	–	−1.43*** (0.39)	−1.28*** (0.42)
Demographic	Rural population (% of the total population)	–	–	–	–	–	1.41 (1.24)	–	0.77 (1.38)
Regional	Region dummies	No	Yes	Yes	Yes	Yes	Yes	Yes	Yes
Temporal	Time dummies	No	Yes	Yes	Yes	Yes	Yes	Yes	Yes
	Constant	9.59 (16.19)	93.55*** (20.52)	124.48 (166.04)	68.24** (26.30)	172.92*** (38.34)	33.28 (62.54)	196.36 (180.77)	175.09 (193.07)
	No. of observations	843	843	843	559	707	842	700	700
	*F*-test	78.22***	44.61***	38.61***	20.61***	38.46***	41.45***	29.80***	26.80***
	*R*-Squared	0.58	0.59	0.58	0.63	0.65	0.60	0.65	0.65

*Note*: 1. *Refers to 10% significance level, **refer to 5% significance level, and ***refer to 1% significance level.

2. Standard Errors are in parenthesis.

Additionally, neighbouring countries’ conflict (*β* = 0.34, *p* < 0.01) was found to be positively associated with MMR (Model 8). Further, it is important to note that the *β*-coefficient for female secondary gross enrolment ratios varied from −1.28 to −1.43 (shown in Models 5 to 8) indicating an inverse relationship between female education and maternal mortality.

**(c) Sub-sample analysis**

We used a sub-sample of cases from African continent, to assess the association between child marriage and RMH outcomes based on conflict exposure in the region. Our results, as shown in Table 5, are consistent with our previous results from the main sample, showing that overall child brides have lower odds of receiving the RMH continuum-of-care compared to women married at 18 and above. Importantly, the child brides exposed to conflict had significantly lower odds (OR: 0.86, *p* < 0.01) of receiving full RMH care than the ones who were never exposed to conflict (OR: 0.95, *p* < 0.01), even after controlling for all the individual, household and other socio-economic variables.

**Table 5.** **Logistic regression estimates: Odds ratios showing effects of child marriage on reproductive and maternal health indicators in conflict versus non-conflict zones in Sub-Saharan Africa** (***N*** **= 282454)**

	Conflict zone					Non-conflict zone			
	At least 1 ANC	4 or more ANCs	4 or more ANCs & institutional delivery	4 or more ANCs & institutional delivery & PNCs		At least 1 ANCs	4 or more ANCs	4 or more ANCs & institutional delivery	4 or more ANCs & institutional delivery & PNCs
Child marriage	0.801*** (0.021)	0.855*** (0.018)	0.853*** (0.020)	0.858*** (0.022)		0.845*** (0.019)	0.968** (0.013)	0.920*** (0.014)	0.948*** (0.016)
Child is male	1.027 (0.018)	1.002 (0.015)	1.031* (0.018)	1.041** (0.019)		0.997 (0.016)	1.010 (0.011)	1.030*** (0.012)	1.041*** (0.012)
Multiple births	1.327*** (0.095)	1.344*** (0.075)	1.708*** (0.107)	1.657*** (0.109)		1.500*** (0.094)	1.275*** (0.048)	1.643*** (0.066)	1.606*** (0.069)
Child’s birth order	0.956*** (0.012)	0.923*** (0.009)	0.844*** (0.011)	0.863*** (0.012)		0.885*** (0.010)	0.870*** (0.007)	0.794*** (0.007)	0.822*** (0.008)
Mother’s age	0.999 (0.002)	1.011*** (0.002)	1.018*** (0.002)	1.019*** (0.002)		0.999 (0.002)	1.016*** (0.001)	1.021*** (0.002)	1.020*** (0.002)
Mother’s age at 1st birth	1.006* (0.004)	1.009*** (0.003)	1.017*** (0.003)	1.013*** (0.004)		1.007** (0.003)	1.003 (0.002)	1.002 (0.002)	1.006** (0.003)
Mother completed primary education	2.331*** (0.073)	1.789*** (0.044)	1.949*** (0.056)	1.890*** (0.062)		2.307*** (0.066)	1.418*** (0.023)	1.444*** (0.026)	1.401*** (0.026)
Mother completed secondary education	3.550*** (0.151)	2.393*** (0.073)	2.694*** (0.087)	2.555*** (0.088)		3.592*** (0.138)	1.863*** (0.040)	2.082*** (0.046)	1.899*** (0.042)
Mother completed higher secondary education	10.503*** (1.399)	5.162*** (0.319)	6.760*** (0.398)	5.178*** (0.280)		7.076*** (1.034)	3.132*** (0.173)	3.496*** (0.186)	3.230*** (0.163)
Household head’s age	0.999 (0.001)	1.002*** (0.001)	1.003*** (0.001)	1.003*** (0.001)		0.999* (0.001)	1.000 (0.001)	1.001 (0.001)	1.001* (0.001)
Household head is male	0.966 (0.033)	0.959 (0.023)	0.954* (0.026)	0.936** (0.028)		0.947* (0.029)	0.947*** (0.015)	0.946*** (0.016)	0.932*** (0.016)
Household size	0.999 (0.004)	0.981*** (0.003)	0.975*** (0.004)	0.979*** (0.004)		1.009* (0.003)	0.999 (0.001)	0.997* (0.002)	0.996* (0.002)
Rural	0.609*** (0.035)	0.773*** (0.026)	0.690*** (0.025)	0.731*** (0.027)		0.585*** (0.025)	0.882*** (0.018)	0.687*** (0.016)	0.756*** (0.018)
Poorer	1.385*** (0.048)	1.200*** (0.034)	1.256*** (0.039)	1.362*** (0.050)		1.296*** (0.033)	1.202*** (0.022)	1.365*** (0.030)	1.373*** (0.031)
Middle	2.069*** (0.086)	1.578*** (0.049)	1.612*** (0.057)	1.824*** (0.075)		1.564*** (0.048)	1.419*** (0.028)	1.675*** (0.041)	1.700*** (0.045)
Richer	2.506*** (0.128)	1.915*** (0.070)	2.133*** (0.085)	2.482*** (0.111)		1.867*** (0.073)	1.663*** (0.037)	2.127*** (0.059)	2.183*** (0.063)
Richest	4.254*** (0.303)	2.599*** (0.117)	3.297*** (0.161)	3.881*** (0.207)		3.304*** (0.204)	2.331*** (0.067)	3.205*** (0.107)	3.132*** (0.108)
Media exposure	1.463*** (0.043)	1.441*** (0.033)	1.464*** (0.040)	1.538*** (0.050)		1.550*** (0.038)	1.236*** (0.020)	1.302*** (0.025)	1.290*** (0.026)
% of households in poorest quintile at provincial level	0.988*** (0.001)	0.991*** (0.001)	0.982*** (0.001)	0.985*** (0.001)		0.993*** (0.001)	0.997*** (0.001)	0.995*** (0.001)	0.997*** (0.001)
Country dummies	Yes	Yes	Yes	Yes		Yes	Yes	Yes	Yes
Year dummies	Yes	Yes	Yes	Yes		Yes	Yes	Yes	Yes
Observations (*n*)	100200	100200	100202	99495		182252	182252	182252	181779

*Note*: Standard errors in parentheses. *p*-values: * *p* < 0.1, ** *p* < 0.05, *** *p* < 0.01.

**(d) Karlson-Holm-Breen (KHB) Decomposition Analysis**

We used KHB decomposition analysis as a mediation analysis to understand the mediating effects of child marriage in case of armed conflicts on RMH outcomes. The KHB method is designed specifically for mediation analysis in nonlinear models such as logistic regression, where coefficients from nested models cannot be compared directly due to rescaling. The KHB technique adjusts for this rescaling, allowing decomposition of the total effect of conflict exposure into the portion that operates directly and the portion that is mediated through child marriage. This provides a statistically valid way to isolate how much of the association between conflict and maternal healthcare utilization is explained by changes in child marriage patterns.

The equation for the analysis is given as:
(5)

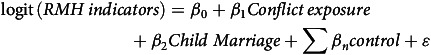




The decomposition components are given as:

Reduced model (Total effects):
(6)

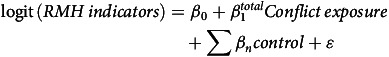




Full model (Direct effects):
(7)

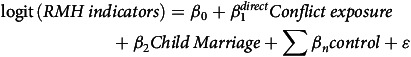




Mediated effects:
(8)

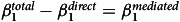




In Equation 5, 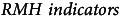 represent the four outcome variables (At least one ANC, four or more ANCs, four or more ANCs & institutional delivery, four or more ANCs & institutional delivery & PNCs). 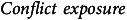 is the main independent variable, and 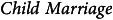 is the mediator. 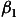 is the total effect of the conflict exposure and 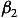 captures the mediating pathway through child marriage. 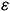 is the error term.

As shown in Table 6, the analysis reveals that child marriage mediates 4.8–9.8% of armed conflict’s negative effects on maternal healthcare utilization in select countries. Conflict reduces ANC visits by 97.5%, with child marriage explaining 4.8% of this effect. For four or more ANC visits (reduced by 51.2% due to conflict), mediation rises to 7.5%. The strongest mediation occurs for institutional delivery (9.8% of conflict’s 50.8% reduction) and postnatal care (9.0% of conflict’s 53.1% reduction). Overall, it shows that while the primary drivers of healthcare disparities remain direct conflict effects (90–95% of total impact), child marriage consistently accounts for a small but significant proportion (*p* < 0.001) across all outcomes. In practical terms, the 4.8–9.8% mediated proportion means that a small but meaningful share of missed ANC visits, reduced facility deliveries, and lower postnatal care among conflict-affected women can be attributed to increased likelihood of child marriage in conflict settings, over and above the direct disruptions caused by violence.

**Table 6.** KHB decomposition analysis: the mediating role of child marriage in the relationship between conflict exposure and reproductive and maternal health indicators (*N* = 452,192)

Outcome variable	Total effect	Direct effect	Mediated effect	% Mediated	*p*-value
At least 1 ANC	−0.975***	−0.928***	−0.047***	4.8	< 0.001
4 or more ANCs	−0.512***	−0.474***	−0.039***	7.6	< 0.001
4 or more ANCs & institutional delivery	−0.508***	−0.458***	−0.050***	9.8	< 0.001
4 or more ANCs & institutional delivery & PNCs	−0.531***	−0.483***	−0.048***	9.0	< 0.001

*Note*: All models control for child marriage as the mediator; *** *p* < 0.001; % Mediated = [(Total Effect - Direct Effect)/Total Effect] × 100; Coefficients represent log-odds from logistic regression models.

## Discussion and conclusion

The elimination of child marriage has long been a priority for improving health and development outcomes, particularly within the framework of RMH, drawing sustained attention from both researchers and policymakers (Fan and Koski, 2022). Child marriage disproportionately exposes women to a range of adverse socio-economic and health consequences, and these vulnerabilities are further exacerbated in catastrophic contexts such as armed conflict, where women face additional demographic, economic, and political insecurities (Singh *et al*., 2022). Despite extensive evidence documenting the harmful effects of child marriage on maternal and reproductive healthcare, its specific implications within armed conflict settings remain insufficiently understood. Moreover, examining the intersection of armed conflict and child well-being is essential for advancing progress toward the United Nations SDGs, particularly SDG 3 (Good Health and Well-being), SDG 5 (Gender Equality), and SDG 16 (Peace, Justice, and Strong Institutions), which collectively underscore the need for accessible healthcare, gender equity, and resilient institutions in conflict-affected environments.

In this context, our study adds to the literature, by examining the association between child marriage and RMH care utilization in conflict versus non-conflict zones using a comprehensive sample of 452,192 women aged 15–49 years based on multi-country datasets over a 15-year period. It investigates the impact of armed conflicts and child marriages as a combined barrier to RMH care utilization.

Our multivariate regression analysis shows that women married before 18 years of age from conflict zones were significantly less likely to access or utilize maternal healthcare. Importantly, despite controlling for various confounding factors, our regression analysis showed that child brides exposed to conflict zones (during the conception year of their child till the interview year) were significantly less likely to utilize maternal health care, relative to women who were not married early or were not exposed to conflicts. Notably, women who were married younger than 18 years (child brides) were significantly less likely to utilize maternal health care during their pregnancy as well as after childbirth, regardless of whether or not they lived in a conflict zone. These findings were further strengthened by the sensitivity analysis. For instance, the inclusion of an additional variable, the interaction of armed conflicts and child marriages, is shown to be statistically significant and associated with a lower likelihood of utilizing maternal healthcare services. Further, the macro-level analysis highlighted that child marriages in armed conflict situations are significantly associated with the MMR of the country. Furthermore, mediation effect findings suggest that interventions must address both conflict-related healthcare disruptions and child marriage pathways and underscores the need for targeted policies in conflict zones to mitigate both direct and indirect barriers to RMH access.

Our results also suggest that child marriage is a significant barrier to access to RMH care. Some possible explanations for the association can be drawn from our descriptive or bivariate results, which showed that a high proportion of child brides have no education, no autonomy over their healthcare decisions, as well as no media exposure. This is in keeping with previous literature depicting child marriage as an important barrier in educational attainment (Field and Ambrus, 2008; Lloyd and Mensch, 2008; Delprato *et al*., 2015; Nguyen and Wodon, 2015), which in turn is further linked with household-level autonomy for women (Jensen and Thornton, 2003; Wodon *et al*., 2016; Male and Wodon, 2018), including healthcare-related decision-making (Osamor and Grady, 2016). Our bivariate results further highlight that child brides have higher a higher proportion of unemployment, possibly attributable to their lower educational status, skills, and lack of relative work opportunities (Soler-Hampejsek *et al*., 2021). Against this backdrop, conflicts can further worsen their vulnerability by destroying essential infrastructure. Conflict situations further increase the incidence of physical and sexual violence and can further restrict the mobility of women. The findings of our study are aligned with previous studies on child marriages and their negative associations with RMH care (Godha *et al*., 2016), viz., pre-natal care (Raj, 2010; Nasrullah *et al*., 2013; Olamijuwon *et al*., 2017; Sekine and Carter, 2019), institutional delivery (Santhya *et al*., 2010; Godha *et al*., 2013; Nasrullah *et al*., 2013; Uddin *et al*., 2019) and post-natal care (Yaya *et al*., 2019).

Based robust empirical evidence, this study demonstrates a greater negative maternal health care consequence of armed conflicts for women married below 18 years than those married 18 years and above. However, we acknowledge the limitations of the study. One of the limitations of this study is the unavailability of a longitudinal or panel dataset, which meant that we were restricted to look at associations instead of establishing causal relationship. Additionally, the cross-sectional design of the DHS data limits the ability to establish temporality between conflict exposure, child marriage, and RMH outcomes. Further, the possibility of reverse causation cannot be ruled out. For instance, limited access to maternal health services or weak health systems may themselves increase household vulnerability, potentially accelerating early marriage as a coping mechanism during periods of insecurity or economic stress (Girls Not Brides, 2020). Communities with chronically poor health service availability may also be at higher risk of conflict-driven displacement, indirectly shaping both marriage practices and health-seeking behaviour (Stark and Ager, 2011). The availability of longitudinal and panel datasets, in future may enable future studies to better establish causal relationships and temporal dynamics.

Secondly, our conflict variable, i.e., battle-related deaths, does not allow us to fully capture conflict intensity in all its dimensions. While it offers a standardized and quantifiable measure, it does not fully reflect the broader spectrum of conflict experiences that may affect health outcomes, such as displacement, sexual and gender-based violence, destruction of infrastructure, or prolonged insecurity. Future studies can benefit from incorporation of more nuanced and multidimensional measures of conflict nature and intensity and employing mixed-methods approaches involving qualitative interviews and participatory research in both conflict and post-conflict settings, which can help uncover the context-specific pathways linking child marriage and RMH outcomes. Leveraging georeferenced DHS cluster data can allow assignment of individual level exposure with a gold standard identification strategy to better identify causal effects of conflict on maternal health outcomes mediating through child marriages. Further studies could also focus on comparative analyses between conflict-affected and stable regions to assess resilience factors, coping strategies, and the role of community health systems in mitigating adverse outcomes.

In conclusion, despite the above-mentioned limitations, the study contributes significantly to both policy and practical implications. Efforts to enhance RMH care in conflict countries must place emphasis on tackling early marriages and assisting adolescent mothers through targeted population and public health policies. By using a pooled sample of microdata from a large number of DHS countries and integrating them with Uppsala conflict data supplemented by macro-data analyses at the global scale, the study also contributes to the literature methodologically. Thus, the study fills the gaps in the child wellbeing literature by shedding light on the status of maternal health care utilization among child married women in armed conflict zones, with a special emphasis on the intersection of conflict and child marriage as a prominent social barrier in accessing maternal healthcare utilization.

To enhance conflict-sensitive maternal healthcare programming, humanitarian actors and governments can adopt mobile outreach clinics and health teams, prioritize adolescent mothers in service planning, invest in community-based birth preparedness planning, and expand the role of local female health workers who may retain access to hard-to-reach populations during insecurity. In other words, integrating adolescent-focused maternal health services into community clinics, leveraging mobile health units to reach displaced populations, and coordinating with local NGOs for outreach can help overcome access barriers. Further, incorporating community-based health education, empowerment programmes, and youth-friendly services can help in reducing both structural and informational barriers that limit adolescent mothers’ access to RMH care. Legal reforms that raise the age of marriage must be accompanied by strong enforcement mechanisms and integrated with service delivery, particularly in conflict zones where institutional structures are weak or eroded. In many humanitarian settings, girls may be married early despite laws due to security fears or economic pressures. Bridging the gap between legal frameworks and frontline maternal care, through school-based services, youth-friendly health initiatives, and community awareness, can reduce the adverse health impacts associated with child marriage.

## Data Availability

All codes and data will be available on request.

## Appendix

**Table A1.** Number of cases (or sample-size) by selected countries in different regions

Region	Country	Number of cases (*n*)	Percentage
East Asia & Pacific	Cambodia	18,261	4.04
	Indonesia	14,951	3.31
	Philippines	14,422	3.19
Europe & Central Asia	Albania	1306	0.29
	Armenia	2160	0.48
	Kyrgyz Republic	3045	0.67
	Moldova	1285	0.28
	Tajikistan	3421	0.76
Latin America & Caribbean	Bolivia	8128	1.80
	Guatemala	8979	1.99
	Guyana	1140	0.25
	Haiti	9084	2.01
	Honduras	8140	1.80
Middle East & North Africa	Egypt	19667	4.35
	Jordan	13389	2.96
	Morocco	4721	1.04
	Yemen	10314	2.28
South Asia	Afghanistan	18984	4.20
	Bangladesh	21319	4.71
	Nepal	4012	0.89
	Pakistan	7398	1.64
Sub-Saharan Africa	Burkina Faso	14275	3.16
	Burundi	4524	1.00
	Cameroon	8656	1.91
	Chad	14030	3.10
	Comoros	1707	0.38
	Congo	5752	1.27
	Congo Democratic Republic	15418	3.41
	Eswatini	1249	0.28
	Ethiopia	7565	1.67
	Gambia	5146	1.14
	Ghana	5357	1.18
	Guinea	7533	1.67
	Kenya	8520	1.88
	Lesotho	7307	1.62
	Liberia	7148	1.58
	Madagascar	1979	0.44
	Mali	6366	1.41
	Mozambique	9640	2.13
	Namibia	1686	0.37
	Niger	13309	2.94
	Nigeria	36703	8.12
	Rwanda	14449	3.20
	Senegal	31714	7.01
	Sierra Leone	6572	1.45
	Togo	4773	1.06
	Uganda	4537	1.00
	Zambia	8113	1.79
	Zimbabwe	4038	0.89
Total (*N*)		452,192	100

**Table A2.** Definitions of the variables used for the macro-level analysis

Variables	Definition
Maternal mortality ratio	The maternal mortality ratio (MMR) is defined as the number of maternal deaths during a given time period per 100,000 live births during the same time period.
Child marriage rate	The proportion of women married below 18 years of age among the women in the age group of 20–24 years
Conflict intensity	Total battle-related deaths per 1000 population in a country in a given period
Neighbouring conflict intensity	Total battle-related deaths per 1000 population in the neighbouring countries of a country in a given period
log gross domestic product per capita (Constant 2010 US$)	Log of sum of gross value added by all resident producers in the economy plus any product taxes and minus any subsidies not included in the value of the products at constant 2010 U.S. dollars divided by the total mid-year population
Poverty headcount ratio at $1.90 a day (2011 PPP)	Percentage of the population living on less than $1.90 a day at 2011 international prices
Female Secondary School Gross Enrolment Ratio (GER)	The ratio of total female enrolment, regardless of age, to the population of the age group that officially corresponds to the secondary level of education
Rural population (% of total population)	Percentage of total rural population out of total population

**Table A3.** Summary statistics of the variables used for the macro-level analysis

**Variables**	**Country**	***n***	**Mean**	**SD**	**Min.**	**Max.**
Maternal mortality ratio	181	904	213.55	316.42	2	2563.50
Child marriage rate	210	979	16.30	17.17	0	81.00
Conflict intensity	217	1,085	1.94	18.83	0	535.35
Neighbouring conflict intensity	217	1,085	9.65	42.72	0	605.72
GDP per capita (constant)	206	994	14277.42	21592.93	189.96	189754.90
Poverty headcount ratio	164	597	16.02	21.92	0	94.10
Female secondary GER	197	817	75.78	32.87	3.20	171.38
Rural population (% of total population)	215	1,074	44.24	23.83	0	92.58
*N*	217	1085				
